# Graph Neural Networks Reveal Candidate Protein Biomarkers Underlying Abdominal Aortic Aneurysm Biology

**DOI:** 10.34133/csbj.0084

**Published:** 2026-05-14

**Authors:** Venkat Ayyalasomayajula, Lotte Rijken, Vivian Waard, Jelmer M. Wolterink, Kak Khee Yeung

**Affiliations:** ^1^ Department of Surgery, Amsterdam University Medical Center (UMC), Amsterdam, The Netherlands.; ^2^ Amsterdam Cardiovascular Sciences, Atherosclerosis and Aortic Diseases, Amsterdam, The Netherlands.; ^3^ Amsterdam Public Health, Digital Health, Amsterdam, The Netherlands.; ^4^ Department of Medical Biochemistry, University of Amsterdam, Amsterdam UMC, Location Academic Medical Center, Amsterdam, The Netherlands.; ^5^ Department of Applied Mathematics, Technical Medical Center, University of Twente, 7522 NB Enschede, The Netherlands.

## Abstract

Abdominal aortic aneurysm (AAA) remains difficult to detect early due to its silent progression and lack of reliable molecular biomarkers. Computational prioritization of disease-associated proteins from protein–protein interaction (PPI) networks offers a scalable alternative to traditional experimental discovery, yet no prior study has applied graph neural network (GNN) architectures to AAA-specific biomarker prediction. We address this gap by developing a positive-unlabeled (PU) learning framework combining message-passing GNNs with PU-specific loss functions to rank candidate AAA-associated proteins across a proteome-wide PPI network, requiring only literature-curated positive labels without plasma samples or experimental validation data. Using 4 PU models on a PPI network of 8,300 proteins and 29,744 interactions, we identified 182 candidate proteins, of which 19 were prioritized through network topology, functional enrichment, and disease relevance. These proteins, including integrins, extracellular matrix regulators, and inflammatory mediators, map to core vascular processes implicated in AAA. Gene Ontology and Kyoto Encyclopedia of Genes and Genomes enrichment supported their mechanistic roles, and transcriptomic and microRNA evidence corroborated several predictions. Notably, COL6A3 has been independently identified as the strongest causal protein signal for AAA in a proteome-wide Mendelian randomization study, providing external validation of our framework. We additionally report 96 novel candidates not retained after enrichment filtering, including 17 predicted independently by all 4 models, representing potential new AAA biology for experimental follow-up. All candidates require prospective experimental validation before clinical application. This work provides a systems-level computational framework for mechanistically grounded AAA biomarker discovery and highlights high-priority candidates for experimental and translational follow-up.

## Introduction

An abdominal aortic aneurysm (AAA) is a life-threatening vascular disease characterized by progressive weakening and dilation of the abdominal aorta [1,2]. Despite its high mortality upon rupture, an AAA often remains asymptomatic during early stages, making timely diagnosis and intervention challenging [1]. Current diagnostic strategies are limited to frequent follow-up of patients with AAA until they reach a diameter threshold for elective repair or become symptomatic [2].

Early detection of AAA-specific protein biomarkers could markedly improve diagnosis, risk stratification, and therapeutic targeting. Traditional biomarker discovery has relied on high-throughput methods such as mass spectrometry and immunoassays to identify differentially expressed proteins between AAA patients and controls [3–5]. However, the complex plasma proteome, dominated by high-abundance proteins like albumin, poses significant barriers to detecting low-abundance, disease-relevant biomarkers [6,7]. Furthermore, these approaches are often time-consuming, are costly, and require extensive validation efforts [8,9].

Computational methods, particularly those leveraging protein–protein interaction (PPI) networks, offer powerful alternatives. Machine learning techniques, such as support vector machines (SVMs) [10,11], random forests [12–14], and neural networks [15,16], have advanced the identification of biomarkers by uncovering complex proteomic patterns, including low-abundance proteins that are often missed by traditional methods [17].

Graph neural networks (GNNs) have introduced a transformative shift in how PPI networks are analyzed for disease association by enabling models to learn directly from the graph structure, capturing both local and global topological dependencies. This allows for the identification of biologically meaningful interactions including indirect or context-specific links that traditional machine learning methods, which rely on handcrafted features, often overlook [18–20].

Despite their potential, applications of GNNs in protein biomarker discovery remain very few, and those that exist often emphasize generic network representations rather than domain-specific biological features (e.g., tissue-specific expression and disease annotations). Moreover, they typically stop at methodological validation and do not evaluate clinically actionable protein outputs, which limits their translational relevance.

Another major challenge in biomarker discovery is the absence of experimentally validated negative protein–disease associations. Most proteins remain uncharacterized, meaning conventional supervised learning cannot be applied because reliable negatives are missing. This motivates positive-unlabeled (PU) learning, designed for settings where only a limited set of positives is available and the remainder is unlabeled [21–26]. PU learning treats the unlabeled set as a mixture of hidden positives and true negatives, enabling discovery of disease-associated candidates from incomplete annotation landscapes [25].

Models such as ProDiGe [22], PEGPUL [23], and PUDI [21] have demonstrated effective strategies for handling unlabeled data, with more recent methods such as C-PUGP [24] incorporating graph propagation heuristics for improved accuracy. However, these approaches are not GNNs; they do not learn node representations through trainable layers, but instead rely on predefined feature vectors (e.g., gene expression profiles and Gene Ontology [GO] terms) [25,26]. As a result, they fail to fully exploit the underlying structure of PPIs, limiting their capacity to model biologically meaningful interdependencies. A direct methodological comparison to the proposed method is provided in Table 1.

**Table 1.** Methodological comparison of PU-GNN with prior disease gene prioritization frameworks. Comparison across 5 dimensions: trainable node embeddings, message-passing GNN architecture, PU-specific loss function, graph structure incorporated in loss, and disease-specific sequence features. Our framework is the only approach combining all 5 components simultaneously. PU-GNN row highlighted in bold.

Method	Trainable node embeddings	Message-passing GNN	PU-specific loss	Graph structure in loss	Disease-specific sequence features
ProDiGe [22]	No	No	Yes	No	No
C-PUGP [24]	No	No	Yes	Yes (propagation)	No
PEGPUL [23]	No	No	Yes	No	No
PUDI [21]	No	No	Yes	No	No
**PU-GNN (ours)**	**Yes**	**Yes**	**Yes**	**Yes**	**Yes**

These limitations highlight a broader gap in AAA biomarker discovery: existing computational approaches do not jointly model PPI topology with incomplete biological labels, preventing them from fully exploiting the structure of protein interaction networks for disease gene prioritization. To address this gap, we developed an integrated framework combining GNNs with PU learning to predict proteins associated with AAA. GNNs learn biologically meaningful node representations from the PPI network, while PU learning infers likely disease-associated proteins from an unlabeled background. By leveraging network structure and semisupervised inference, our approach uncovers AAA biomarker candidates overlooked by traditional methods.

## Methods

### PPI graph

A PPI network was constructed using high-confidence interactions from the STRING (Search Tool for Retrieval of Interacting Genes/Proteins) database (v12.0, accessed 2024 May 22), with experimental interactions from BioGRID (version 4.4.212, accessed 2024 May 22) used for cross-validation of interaction evidence. Only STRING interactions with a confidence score above 700 were retained. Since STRING and BioGRID do not annotate interactions with tissue or disease-specific context, interactions were analyzed in a unified network, which may overlook context-dependent dynamics. The largest connected component was retained for analysis; smaller components and isolated nodes were excluded to ensure effective message-passing during GNN training.

**Fig. 1.**
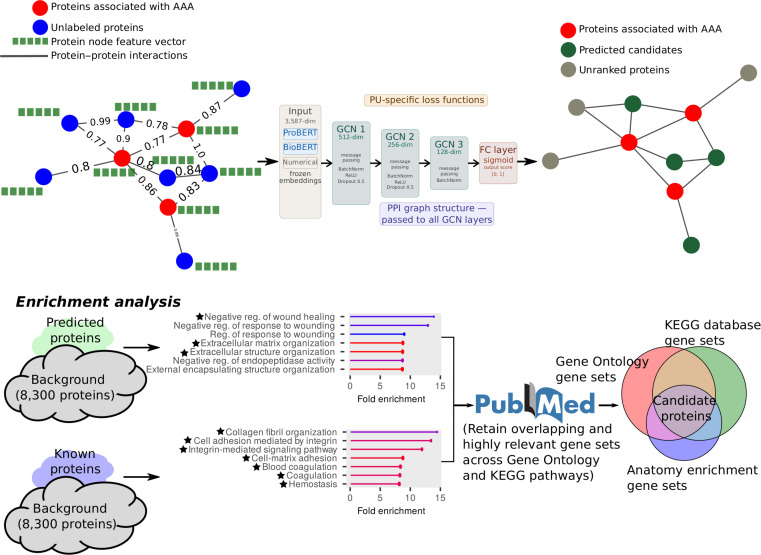
Schematic overview of the integrated PU-GNN framework for abdominal aortic aneurysm (AAA) biomarker discovery.

Protein node characterization was achieved by integrating annotations from NextProt (overview), UniProt (functional descriptions, amino acid sequences, and molecular weights), and Kyoto Encyclopedia of Genes and Genomes (KEGG) (nucleotide sequence data). These features provided insights into the biological roles and interaction dynamics of proteins. Protein identifiers were harmonized to UniProt IDs for consistency across datasets, ensuring reliable network modeling. Embeddings were generated using ProBERT for protein sequences and BioBERT for textual descriptions (overview and function). We incorporated 3 numerical features: molecular weight, amino acid sequence length, and nucleotide sequence length. Each protein node was represented by a concatenated embedding of 3,587 dimensions, consisting of: BioBERT embeddings (2 × 768) for both protein function and description, ProBERT embeddings (2 × 1,024) for amino acid and nucleotide sequences, and 3 numerical features. Disease labels for AAA were assigned to 241 proteins identified through a systematic literature search of untargeted proteomics and mass spectrometry panel studies comparing AAA patients to non-aneurysmal controls. Studies profiling both plasma and aortic tissue were included. Inclusion criteria were as follows: (a) human AAA samples; (b) case–control design with non-aneurysmal controls; (c) untargeted or panel-based protein quantification; and (d) differential protein expression reported. Proteins associated exclusively with other cardiovascular conditions without AAA-specific evidence were excluded. In the constructed PPI network, a total of 8,300 proteins and 29,744 interactions were included for analysis, of which 241 carry positive AAA labels and 8,059 constitute the unlabeled candidate pool. An overview of the full framework is presented in Fig. 1.

### GNN architecture and training

A 3-layer graph convolutional network (GCN) architecture was utilized for predicting protein–disease associations. The GCN is a widely used instantiation of the broader GNN family, in which node representations are updated by aggregating and transforming features from immediate graph neighbors. Each GCN layer aggregated information from neighboring nodes in the PPI network, capturing both local and global dependencies. Batch normalization was applied after each layer to stabilize training, and dropout (*P* = 0.5) was employed to reduce overfitting and enhance generalization. A ReLU (rectified linear unit) activation function introduced nonlinearity after each layer. The model was trained using PU objectives rather than a standard supervised loss. As explicit negatives are unavailable, standard supervised losses cannot be applied directly. PU training addresses this by decomposing the objective into a positive risk term, estimated on the labeled positives, and a surrogate negative risk term, estimated from the unlabeled pool. Different PU loss formulations vary in how they handle this surrogate risk: some assume a class prior to weight the positive contribution in the unlabeled set, while others apply correction strategies to reduce bias from hidden positives. In this study, we implemented and compared multiple PU-oriented loss functions that adapt the classification objective in slightly different ways; the exact definitions are provided below.

All protein sequence embeddings (ProBERT) and functional text embeddings (BioBERT) were used in frozen inference mode. Embedding weights were not updated during model training; the language model parameters were fixed, and embeddings were precomputed once and stored as static feature vectors prior to all experiments. Consequently, the 3,587 embedding dimensions do not constitute trainable parameters in the model optimization. The effective learnable parameter space is confined to the 3 GCN layers and the final fully connected layer, substantially reducing overfitting risk relative to what the raw feature dimensionality might suggest. All experiments used random seed 42. Training was performed on an NVIDIA H100 NVL GPU (47GB MIG partition) on the Helios HPC cluster. Code is available at https://github.com/RadhakrishnaAyyalasomayajula/AAA-PU-GNN.

The positive class prior probability was specified as follows across the 4 PU loss functions: nonnegative PU (nnPU) used a fixed prior of 0.10, reflecting a conservative estimate of the proportion of AAA-associated proteins in the full proteome; DistPU used a fixed prior of 0.10; PUGNN used a dual prior of 0.5 during the warm-up phase and 0.1 during the main training phase; and GRAB estimated the prior dynamically per epoch as the proportion of unlabeled nodes exceeding the 0.5 sigmoid threshold, capped at a maximum of 0.05. These values are consistent with the approximate background prevalence of known AAA-associated proteins in the network (241/8,300 approximately 0.029) and account for the expected proportion of unknown positives in the unlabeled pool. Model outputs are raw sigmoid scores and should be interpreted as ranking scores rather than calibrated probability estimates of AAA association. Adam optimizer, with a learning rate of 0.001, was used for training over 250 epochs, and early stopping was applied if validation performance, measured as recall on the spy set, did not improve for 10 consecutive epochs. Learning curves for all 4 models across 5 folds are provided in Fig. S1.

### Selection of PU learning loss functions

In this study, the model’s task is to predict whether a protein is associated with AAA. We employed 4 loss functions specifically designed for PU learning. Each of these losses adapts the training objective to learn from labeled positives and unlabeled data, but they differ in how the unlabeled risk is estimated and corrected. In standard supervised binary classification, the risk is defined over positives (P) and negatives (N) as:

$$\begin{array}{c} R_{\sup}\left(f\right)=\\ \pi_{p}E\left\{x∼P\right\}\left[\ell \left(f\left(x\right),1\right)\right]+\left(1-\pi_{p}\right)E\left\{x∼N\right\}\left[\ell \left(f\left(x\right),0\right)\right] \end{array}$$ (1)

where *f*(*x*) is the model prediction for sample *x*, *ℓ* is the pointwise classification loss (binary cross-entropy), 𝑃 and 𝑁 denote the distributions of positive and negative samples, $\pi_{p}$ is the prior probability of positives in the population, and $E\;x∼D\;\left[\cdot \right]$ denotes the expectation of the loss over samples drawn from distribution 𝐷. In PU learning, negatives are not available, so the negative risk is estimated from the unlabeled set (U) and corrected for the fact that some positives may also be hidden within U. A generalized PU risk can be expressed as:

$$\begin{array}{c} R_{\mathrm{PU}\left(f\right)}=\pi_{p}E\left\{x∼P\right\}\left[\ell \left(f\left(x\right),1\right)\right]\\ \;+\left(E\left\{x∼U\right\}\left[\ell \left(f\left(x\right),0\right)\right]-\pi_{p}E\left\{x∼P\right\}\left[\ell \left(f\left(x\right),0\right)\right]\right) \end{array}$$ (2)

Here, the first term is the positive risk, and the second term is a surrogate negative risk estimated from U and corrected for hidden positives. Different PU losses vary in how they handle or correct this surrogate negative risk, but all share this decomposition into positive and unlabeled terms. The 4 PU loss functions used in our experiments are summarized below:

• nnPU: It introduces a nonnegative correction to the PU risk estimator, preventing the model from becoming biased when hidden positives are present in the unlabeled set. This stabilizes training and reduces overfitting, enabling the use of flexible models such as deep neural networks with limited positive labels [27].
• Dist-PU: It aligns predicted label distributions with expected class distributions, reducing confirmation bias. It employs entropy minimization and mix-up regularization to improve consistency across unlabeled samples [28].
• PU-GNN: It extends nnPU by incorporating message-passing layers over the PPI graph, enabling propagation of positive signal to indirect neighbors while controlling false-positive bias. This enhances supervision and alignment for node classification tasks where only positive labels are known [29].
• GRAB: It is a graph-based PU method that iteratively refines node labels through belief propagation, eliminating the need for an assumed prior on the proportion of positives [30].

Each model trained with one of these 4 PU losses produces its top 100 predicted candidate protein–disease associations. Because the sets of predictions differ across models, we take the union of the top 100 from each model to obtain a consolidated pool of candidate associations, which is subsequently pruned and analyzed through enrichment analysis (see the “Quantitative evaluation” section).

### Quantitative evaluation

To evaluate model performance in the PU setting, we employed 5-fold cross-validation applied to the known AAA-associated proteins set rather than the full dataset. In each fold, known AAA-associated proteins (positives) were split into a training subset (80%) and a spy subset (20%). The spy subset serves a distinct purpose from standard model evaluation: spy proteins are biologically positive but their labels were withheld during training and they were included in the unlabeled pool, thereby simulating a PU scenario in which some true positives are unlabeled. Model performance was then evaluated by assessing recovery of these withheld positives. Early stopping was applied if performance on the spy set did not improve for 10 consecutive epochs.

The evaluation metric was recall (sensitivity), defined as:

$$\text{Recall}=\frac{\mathrm{TP}}{\left(\mathrm{TP}+\mathrm{FN}\right)}$$ (3)

where TP denotes spy positives correctly identified and FN denotes spy positives not recovered by the model. Recall was chosen because it directly measures the model’s ability to recover hidden positives within the unlabeled pool, which is the central challenge in PU learning. Although recall alone can be trivially maximized, rank-based evaluation and consensus filtering ensure that high recall reflects meaningful enrichment.

Alternative criteria based on the F1 score have been proposed in the PU literature [31]. Under the SCAR assumption (selected completely at random; see the “Limitations and future directions” section for discussion of this assumption’s validity), recall can be estimated from PU data as $r=\mathrm{Pr}\left(\hat{y}=1|s=1\right)\;$, but precision cannot be obtained directly because the unlabeled pool contains an unknown mixture of positives and negatives. As a result, the true F1 cannot be computed. In this work, we report both recall and surrogate F1, which approximates the F1 score by using recall and the prior probability of positives (estimated from the data) to infer a surrogate measure of precision. Recall is directly measurable from the spy set, and surrogate F1 provides a complementary perspective consistent with standard classification metrics.

For each loss function, recall was computed on the spy set in every fold, and results are reported as mean ± standard deviation across folds. Model performance is thus summarized directly by recall values, without further statistical testing, since cross-validation folds are not independent samples.

Prior sensitivity analysis across *π_p_* = 0.01 to 0.30 for nnPU, DistPU, and PUGNN showed mean spy-set recall increasing with prior value but stable top 100 union composition across all configurations and models, demonstrating robustness to prior misspecification (Table S3).

To assess sensitivity to the spy proportion, we evaluated all 4 models across 6 spy-set sizes ranging from 5% to 30% (Table S4). Performance was broadly consistent across all models and spy proportions, with mean recall at the 20% proportion used in the main analysis ranging from 0.53 (nnPU) to 0.83 (PUGNN). The composition of the top 100 union candidate set remained stable across all configurations and model variants, demonstrating that the final candidate list is not critically dependent on the spy proportion chosen.

To evaluate whether the models rank true positives near the top of the prediction list rather than throughout, we report Recall@K as a ranking surrogate metric. For each model, Recall@K measures the proportion of the 19 final validated candidate proteins recovered within the top K predictions across all 8,059 unlabeled proteins. This directly addresses the concern that high recall could reflect diffuse ranking rather than meaningful enrichment at the top of the list. Recall@K results across all 4 models and all baseline comparators are reported in the “Model performance” section.

### Biological enrichment-based refinement

Enrichment analysis was performed on 2 protein lists using the full 8,300-protein PPI network as background, not restricted to the 241 known AAA-associated proteins. Categories considered included anatomical expression, GO biological process (BP), molecular function (MF), cellular component (CC), and KEGG pathways. Statistical significance was assessed using a hypergeometric test (equivalent to Fisher’s exact test), comparing the observed overlap with random sampling from the full network. *P* values were corrected using the Benjamini–Hochberg procedure. High-confidence categories were retained with the following thresholds: FDR ≤ 0.001, fold enrichment > 1, foreground count ≥ 5, and background count ≤ 500.

Enrichment was applied post hoc to avoid biasing the model with prior knowledge. GO/pathway annotations were excluded as node features to prevent information leakage and ensure that the model discovered associations from the PPI network and sequence data.

Enriched terms were assessed for relevance to AAA by searching PubMed for each term paired with “abdominal aortic aneurysm”, retaining only those with documented associations. The final protein list was refined by taking the intersection across all enrichment categories, integrating tissue expression, GO BP, GO MF, GO CC, and KEGG pathways. This strategy enhanced the reliability and interpretability of the protein–disease associations.

## Results

### Model performance

The performance of the model was assessed using 5-fold cross-validation, with recall as the primary metric for both the 80% training set and the 20% spy set. On the spy set, PU-GNN achieved the highest recall (0.83 ± 0.03), followed by GRAB (0.80 ± 0.04), nnPU (0.78 ± 0.05), and Dist-PU (0.69 ± 0.06). Fold-level comparisons indicated that PU-GNN performed significantly better than Dist-PU (*P* < 0.01) and nnPU (*P* = 0.043), while the difference with GRAB did not reach statistical significance (*P* = 0.12).

For each of the 4 PU loss functions, we ranked all proteins by their recall-weighted prediction probability, where fold-level probabilities were averaged using recall as weights so that higher-recall folds contributed more strongly to the final score. From each model, the top 100 ranked proteins were retained. Taking the union of these 4 sets produced 182 unique proteins, which formed the candidate pool for downstream refinement.

To assess whether models rank true positives near the top rather than throughout, we evaluated Recall@K against the 19 final validated proteins across 8,059 unlabeled proteins. At *K* = 200 (top 2.5% of the proteome), PUGNN, nnPU, GRAB, and DistPU recovered 89%, 84%, 84%, and 84% of the 19 final proteins, respectively, confirming systematic enrichment at the top of the ranked list. At *K* = 100, GRAB and DistPU recovered 84% and 79%, respectively, while PUGNN recovered 47% at *K* = 100 but reached 89% by *K* = 200.

Three non-PU baselines were evaluated under identical conditions. The RWR (random walk with restart) baseline recovered 16% at *K* = 100 and 37% at *K* = 200. The feature-only multilayer perceptron (MLP) recovered 21% at *K* = 100 and 26% at *K* = 200. The graph-only GCN ablation recovered 53% at *K* = 100 and 63% at *K* = 200. All 4 PU-GNN models outperformed all 3 baselines at every threshold, demonstrating independent contributions of both graph message-passing and molecular embeddings to prediction performance. Full Recall@K results are reported in Table 2.

**Table 2.** Recall@K comparison across PU-GNN models and non-PU baselines. Proportion of the 19 final validated candidate proteins recovered within the top K predictions across 8,059 unlabeled proteins. PU-GNN models shown in bold.

Model	Type	Recall@50	Recall@100	Recall@200	Recall@500
**PUGNN**	**PU-GNN**	**0.26**	**0.47**	**0.89**	**0.95**
**nnPU**	**PU-GNN**	**0.58**	**0.74**	**0.84**	**0.89**
**GRAB**	**PU-GNN**	**0.68**	**0.84**	**0.84**	**0.95**
**DistPU**	**PU-GNN**	**0.74**	**0.79**	**0.84**	**0.89**
RWR	Baseline	0.16	0.16	0.37	0.89
MLP	Baseline	0.16	0.21	0.26	0.42
GCN (graph-only)	Baseline	0.42	0.53	0.63	0.84

RWR, random walk with restart seeded on 241 known AAA proteins; MLP, feature-only baseline using same embeddings with nnPU loss but no graph structure; GCN (graph-only), topology-derived features only, no language model embeddings

Prior sensitivity analysis across $\pi_{p}=0.01$ to 0.30 for nnPU, DistPU, and PUGNN showed mean spy-set recall increasing with prior value, from 0.42 ± 0.15 at *π_p_* = 0.01 to 0.84 ± 0.08 at $\pi_{p}$ = 0.30 for nnPU. The composition of the top 100 union set remained stable across all configurations and models, confirming robustness to prior misspecification (Table S3).

### Candidate protein associations

From the 182 unique proteins identified via model predictions (“Model performance” section), we applied the multistep enrichment-based refinement pipeline described in Methods (Fig. 1). Of these, 163 proteins did not meet the enrichment overlap criteria and were removed, yielding 19 high-confidence AAA candidate proteins supported by network topology, multimodel PU predictions, enrichment overlap, and external literature evidence. A comparison of all 182 predicted proteins before and after enrichment filtering is provided in Table S2.

**Fig. 2.**
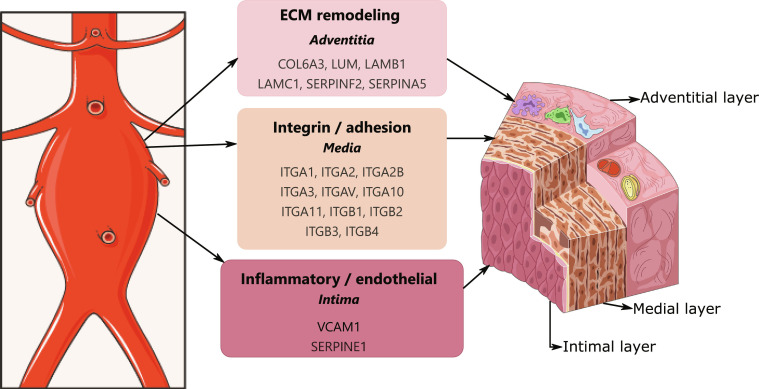
Network visualization of the final 19 candidate proteins. Two proteins (SERPINF2 and SERPINA5) appear as isolated nodes because no interactions with the other candidates passed the STRING confidence threshold of 0.7.

GO enrichment of the 19 candidates revealed significant processes including cell adhesion, integrin-mediated signaling, and extracellular matrix (ECM) organization, while KEGG pathway analysis linked them to ECM–receptor interaction, focal adhesion, and PI3K-Akt signaling, all central to vascular integrity and aortic remodeling. The 19 candidate proteins, their functional annotations, classification, cell type expression, detection modality, and prior AAA evidence are summarized in Table 3. These 19 proteins had an average predicted probability of 0.92 ± 0.03, corresponding to sigmoid outputs aggregated across folds using recall weighting. The spatial distribution of the 19 candidate proteins across the aortic wall is illustrated in Fig. 2, with proteins grouped into 3 biologically coherent modules: inflammatory/endothelial mediators in the intima, integrin/adhesion receptors in the media, and ECM remodeling proteins in the adventitia. Of note, SERPINF2 and SERPINA5 show no interactions with the other 17 candidates at the STRING confidence threshold of 0.7, though both connect to other proteins in the full network outside the final candidate set.

**Table 3.** Candidate AAA-associated proteins identified by the PU-GNN framework. The 19 final candidate proteins are split into Section A (14 proteins with prior AAA proteomics evidence) and Section B (5 proteins for which this study provides the primary computational evidence). Classification: Structural = ECM structural components; Enzymatic = proteolytic regulators; Inflammatory = immune/endothelial mediators; Adhesion = integrin family. Cell types derived from published single-cell RNA sequencing and Human Protein Atlas data [70]. Detection modality indicates experimental approaches used in cited studies. AAA Evidence refers to aortic disease context in which protein has been reported.

Gene	UniProt ID	Protein name	Function	Classification	Cell type (AAA)	Detection modality	AAA evidence	Ref.
**Section A: Established candidates—prior AAA proteomics evidence**								
SERPINE1	P05121	PAI-1	Serine protease inhibitor; fibrinolysis regulation	Enzymatic	VSMCs, endothelium, macrophages	ELISA, MS, WB	AAA tissue, plasma	[43–45]
SERPINF2	P08697	Alpha-2-antiplasmin	Serine protease inhibitor; plasmin inhibition	Enzymatic	Plasma, liver	ELISA, WB	AA, AD, AAA	[46,71]
LUM	P51884	Lumican	ECM proteoglycan; collagen fibril organization	Structural	Fibroblasts, VSMCs	MS (iTRAQ, SWATH)	TAA, AAD	[47,48]
VCAM1	P19320	VCAM-1	Leukocyte adhesion; endothelial activation	Inflammatory	Endothelium	qRT-PCR, IHC	IA, AAA, TAAD	[53,56,57]
LAMB1	P07942	Laminin B1	Basement membrane; cell attachment	Structural	Endothelium, VSMCs	IHC, RT-PCR, WB	ARA	[72,73]
LAMC1	P11047	Laminin C1	Basement membrane; cell migration	Structural	Endothelium, epithelium	IHC	ARA	[72,74]
COL6A3	P12111	Collagen VI alpha-3	ECM structural integrity; cell–matrix adhesion	Structural	Fibroblasts, VSMCs	MS (MR-based)	AAA (MR)	[75]
ITGA1	P56199	Integrin alpha-1	Laminin/collagen receptor; cell–matrix adhesion	Adhesion	VSMCs, fibroblasts	WGCNA, bioinformatics	AAA	[55]
ITGA2	P17301	Integrin alpha-2	Collagen/laminin receptor; platelet adhesion	Adhesion	Platelets, VSMCs	WB, proteomics	TAA, AAA	[32,76]
ITGA2B	P08514	Integrin alpha-IIb	Fibronectin/fibrinogen receptor; platelet activation	Adhesion	Platelets	Proteomics	IA, Type A AAD	[52,77]
ITGAV	P06756	Integrin alpha-V	Vitronectin receptor; efferocytosis	Adhesion	Macrophages, VSMCs	IHC, WB	TAD, AAA	[66]
ITGB1	P05556	Integrin beta-1	ECM receptor; SMC adhesion and survival	Adhesion	VSMCs, macrophages	WB, IHC	AAA, TAA, AAD	[67,68]
ITGB2	P05107	Integrin beta-2	Complement receptor; immune cell adhesion	Inflammatory	Macrophages, neutrophils	MS, WB	TAA, TAAD, AAD, AAA	[53,54,78]
ITGB3	P05106	Integrin beta-3	Vitronectin receptor; VSMC phenotypic switch	Adhesion	VSMCs, platelets	iTRAQ, SWATH-MS	IA, AAA	[49–51]
**Section B: Computational candidates—primary evidence from this study**								
SERPINA5	P05154	SERPINA5	Serine protease inhibitor; extravascular proteolysis	Enzymatic	Plasma, liver	Limited	N/A	N/A
ITGA3	P26006	Integrin alpha-3	Fibronectin/laminin receptor; cell invasion	Adhesion	Epithelium, fibroblasts	MS (TMT)	AAD	[58]
ITGA10	O75578	Integrin alpha-10	Collagen receptor; cartilage/vascular matrix	Adhesion	Chondrocytes, VSMCs	ML-based feature gene	AAA	[59]
ITGA11	Q9UKX5	Integrin alpha-11	Collagen receptor; ECM biosynthesis	Adhesion	Fibroblasts, plasma cells	Genomic analysis	IA, AAA	[79]
ITGB4	P16144	Integrin beta-4	Laminin receptor; hemidesmosome structure	Adhesion	Epithelium, endothelium	Network analysis	Limited	N/A

AAA, abdominal aortic aneurysm; TAA, thoracic aortic aneurysm; AAD, acute aortic dissection; TAAD, thoracic aortic aneurysm and dissection; IA, intracranial aneurysm; ARA, aortic root aneurysm; AD, aortic dissection; VSMCs, vascular smooth muscle cells; MS, mass spectrometry; IHC, immunohistochemistry; WB, Western blot; MR, Mendelian randomization; ML, machine learning

To complement the 19 high-confidence candidates, we separately analyzed proteins predicted in the top 100 by at least 2 of the 4 independent models but not retained after enrichment filtering. This novel-module set comprises 96 proteins, of which 17 were independently predicted by all 4 models, including PCYOX1, GPNMB, CCN2, APOF, F13B, PMEL, HPR, APOL1, ITGA5, APOA4, APOC4, ITGB6, ITGA7, ITGA6, ZP2, TMSB10, and LAMA5. These proteins represent the highest-confidence computational predictions outside established AAA biological axes and are provided in Table S1 for hypothesis-driven experimental follow-up.

### Functional enrichment analysis of candidate proteins

**Fig. 3.**
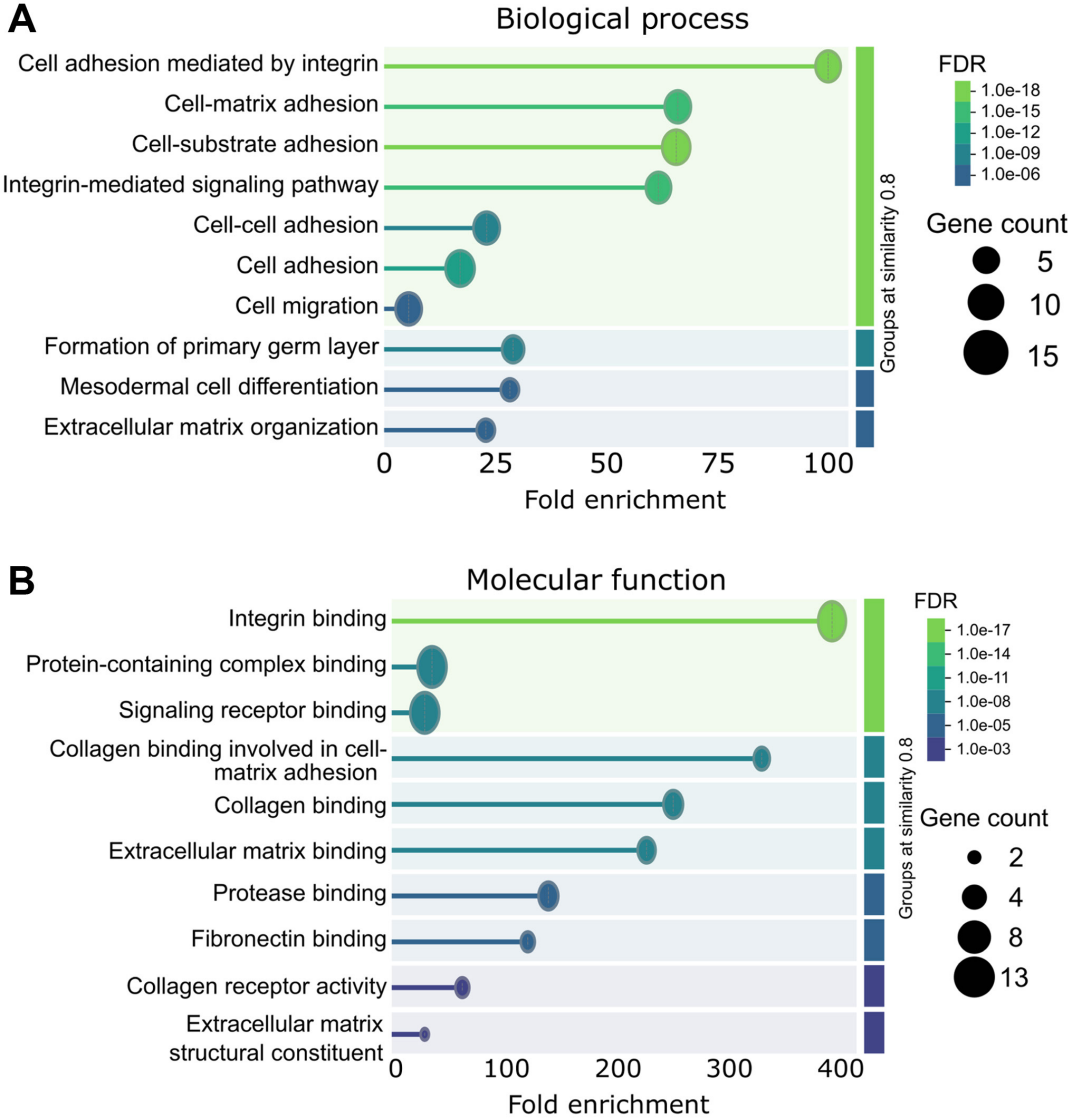
Enrichment analysis of Gene Ontology (GO) terms for the final 19 candidate proteins. (A) The top 10 enriched GO biological process (BP) terms, highlighting the most relevant processes associated with these proteins. (B) The top 10 enriched GO molecular function (MF) terms, illustrating the key functions that these proteins are involved in.

**Fig. 4.**
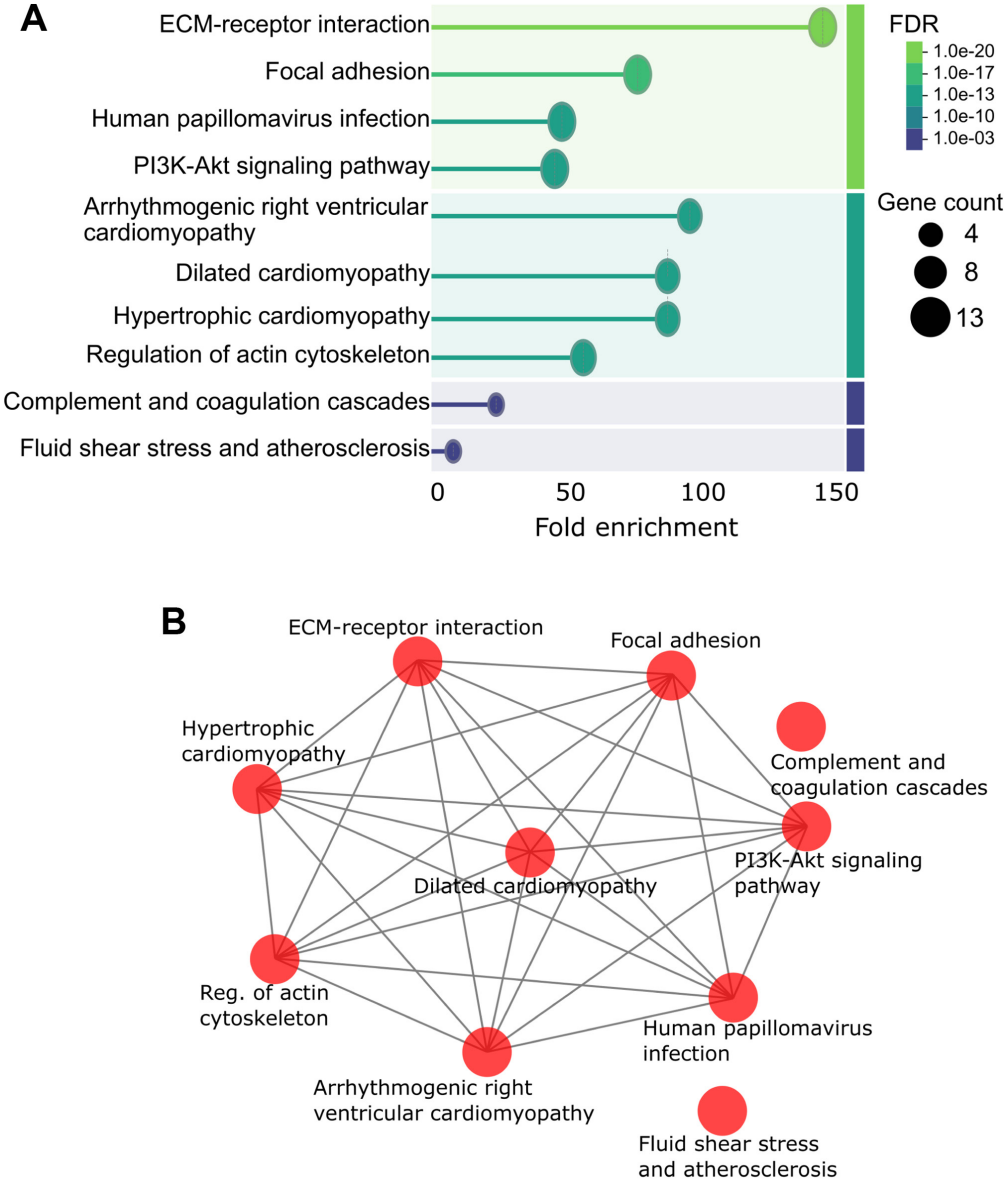
Enriched Kyoto Encyclopedia of Genes and Genomes (KEGG) pathway analysis for the final 19 candidate proteins. (A) Lollipop plot depicting the enriched KEGG pathway terms, highlighting the most significantly enriched pathways. (B) Network plot illustrating the interactions between the enriched KEGG pathways, showcasing the relationships among the identified pathways.

To assess biological relevance, enrichment analysis was performed on the 19 candidate proteins using the full PPI network as background. Within the GO BP category, key enriched terms included cell adhesion (GO:0007155), integrin-mediated signaling (GO:0007229), cell–matrix adhesion (GO:0007160), and ECM organization (GO:0030198), all central to AAA pathophysiology (Fig. 3A). In the MF category, the strongest association was with integrin binding (GO:0005178), alongside collagen binding, ECM binding, fibronectin binding, and protease binding, reflecting key mechanisms in ECM remodeling (Fig. 3B). KEGG pathway analysis identified ECM–receptor interaction (hsa04512), focal adhesion (hsa04510), PI3K-Akt signaling (hsa04151), complement and coagulation cascades (hsa04610), and fluid shear stress and atherosclerosis (hsa05418) as the most relevant enriched pathways, visualized in Fig. 4.

### Contextual enrichment analysis of ensemble proteins: GO and KEGG subnetworks

**Fig. 5.**
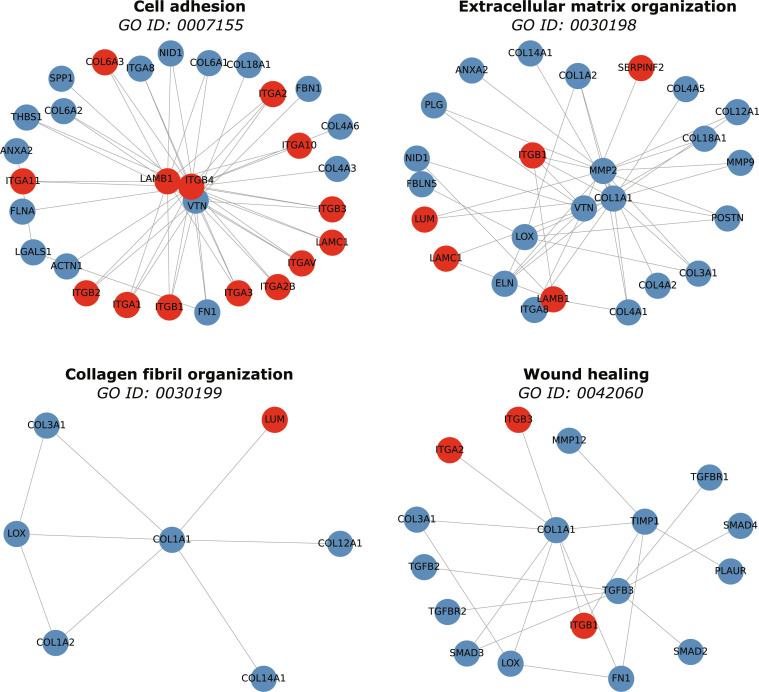
Subnetworks extracted from the PPI graph for 4 enriched GO biological process terms: cell adhesion, extracellular matrix organization, collagen fibril organization, and wound healing. Each panel shows known AAA-associated proteins (blue) and predicted candidate proteins (red) annotated to the given term, connected by high-confidence STRING/BioGRID interactions (gray edges).

**Fig. 6.**
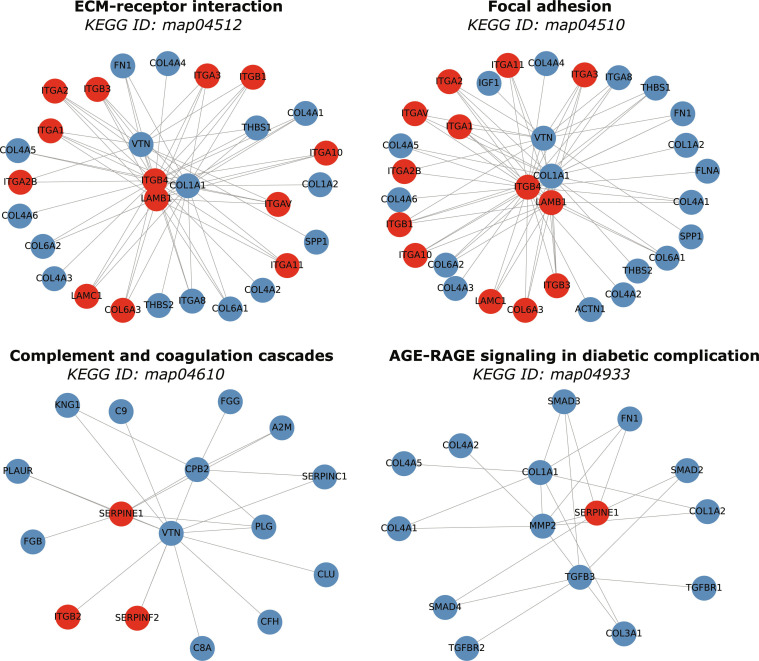
Subnetworks extracted from the PPI graph for 4 enriched KEGG pathways: ECM–receptor interaction, focal adhesion, complement and coagulation cascades, and AGE-RAGE signaling in diabetic complications. Node colors distinguish known AAA-associated proteins (blue) from predicted candidates (red), and edges represent high-confidence protein–protein interactions (gray).

Enrichment analysis of the combined ensemble of 241 known and 19 candidate proteins highlighted cell adhesion, ECM organization, collagen fibril organization, complement and coagulation cascades, platelet activation, AGE-RAGE signaling in diabetic complications, and regulation of actin cytoskeleton as key enriched terms. Subnetwork visualization (Figs. 5 and 6) shows how candidates embed within established AAA modules: integrins (ITGA1, ITGA2, ITGB1, ITGB3, and ITGB4) appear alongside collagens and laminins in ECM–receptor interaction and actin cytoskeleton regulation contexts, while SERPINE1, SERPINF2, and SERPINA5 cluster within complement and coagulation cascades, indicating that candidates both reinforce existing biological modules and provide new links within well-validated AAA processes.

### Tissue enrichment

Tissue enrichment analysis was performed using STRING, with the entire protein network as the background. Significant associations (FDR ≤ 0.001) were identified for key anatomical structures relevant to AAA, including the vascular system (UBERON:0007798), cardiovascular system (UBERON:0004535), heart (UBERON:0000948), blood vessel (UBERON:0001981), bone marrow cells (UBERON:0002092), plasma cells (UBERON:0000786), and aorta (UBERON:0000947). The number of proteins associated with each tissue ranged from 5 to 16, indicating strong tissue-specific expression patterns among the candidate proteins.

### Network parameters of candidate proteins

We further analyzed the network properties of the 19 candidate proteins in the full PPI graph (STRING confidence ≥ 0.7). For each protein, degree centrality, betweenness centrality, clustering coefficient, and the ratio of known-to-unknown AAA-associated neighbors were computed.

Degree centrality ranged from very low (SERPINA5: 2 connections) to high (ITGB4: 72 connections). Integrins such as ITGB1 (15), ITGB2 (13), and ITGAV (11) exhibited substantial degrees, reflecting their established roles as major network hubs. Betweenness centrality, which highlights bridging nodes, was highest for ITGB4 (1.0 × 10^−2^), SERPINE1 (5.0 × 10^−3^), and ITGB2 (1.8 × 10^−3^), indicating that these proteins serve as connectors between network regions. Clustering coefficients revealed variation in neighborhood density: proteins such as LUM (0.80), ITGA10 (0.83), and ITGA11 (0.80) formed dense local clusters, while SERPINA5 (0.00) and ITGB4 (0.05) showed sparse local structure.

The annotation of neighbors revealed further distinctions. SERPINE1 (19/51, ratio 0.37) and ITGB4 (15/72, ratio 0.21) had many known AAA-associated neighbors, whereas proteins such as VCAM1 (0/9) and SERPINA5 (0/2) had none. ECM-associated proteins generally showed higher clustering, while proteins linked to smooth muscle cell (SMC)-related processes (e.g., SERPINE1 and VCAM1) occupied intermediate positions with mixed neighborhood compositions. Several proteins, including SERPINA5 and CNTN2, had few or no adjacent known AAA-associated nodes, placing them in peripheral or sparsely connected regions of the network.

## Discussion

### Key findings

This study presents the first application of message-passing GNN architectures combined with PU-specific loss functions to AAA biomarker prediction. Four complementary PU-GNN models operating on a proteome-wide PPI network identified 19 high-confidence candidate proteins through consensus prediction and enrichment-based refinement. While PU-GNN achieved the highest spy-set recall among the 4 models, differences between models were modest, and the primary strength of the framework lies in the ensemble consensus approach rather than the superiority of any single model variant.

The 19 final candidates include 14 proteins with prior AAA proteomics evidence and 5 proteins—SERPINF2, SERPINA5, ITGA3, ITGA10, and ITGB4—for which our framework provides the primary computational evidence (Table 3). An additional 96 novel-module proteins predicted by at least 2 models but outside established enrichment axes, including 17 predicted by all 4 models independently, represent an exploratory discovery set for hypothesis-driven follow-up.

Independent cross-referencing against proteome-wide Mendelian randomization of AAA [32,33] identified 6 candidates with published causal genetic evidence: COL6A3, ITGA1, ITGB1, LAMB1, LUM, and VCAM1. Notably, COL6A3 represents the strongest causal MR signal for AAA [32], providing direct independent validation of our computational predictions by an orthogonal causal inference approach operating on an entirely independent dataset.

Network-level analysis further shows that the 19 candidates occupy diverse structural roles in the PPI graph. Hub proteins such as ITGB4 and SERPINE1 link into central vascular modules enriched with known AAA-associated proteins, while ITGA10, ITGA11, and SERPINA5 are positioned in peripheral regions with few AAA-annotated neighbors, suggesting they represent underexplored aspects of AAA biology. This heterogeneity underscores the value of integrating molecular embeddings with graph-based learning rather than relying on network topology alone. Together, these findings establish PU-GNN-based proteome prioritization as a viable computational strategy for AAA biomarker discovery, complementing experimental approaches and providing a ranked candidate list for prospective validation in clinical cohorts.

### Core mechanisms: ECM dysregulation and network-driven pathology

The enrichment profile of the 19 candidates points to ECM dysregulation and integrin-mediated signaling as central pathological axes in AAA. Integrins like ITGA3 and ITGB2 are central to cell–matrix adhesion and actively regulate fibroblast and SMC behavior in response to mechanical and inflammatory stimuli [34–36], processes whose disruption drives aortic wall weakening [37]. The enrichment of coagulation and complement cascade pathways further implicates inflammatory amplification in vascular damage [38], while PI3K-Akt pathway involvement suggests an additional regulatory axis governing SMC fate and ECM maintenance with potential therapeutic relevance [39].

Among the 19 candidates, COL6A3, LUM, LAMB1, and LAMC1 directly participate in ECM structural integrity and collagen organization, while SERPINE1 and SERPINF2 regulate proteolytic balance, together representing a coherent ECM remodeling module with direct relevance to aortic wall weakening in AAA.

Notably, pathway analysis reveals mechanistic convergence between AAA and broader cardiovascular pathologies, including cardiomyopathies, atherosclerosis, and shear stress responses [40], through shared drivers of vascular dysfunction. This overlap is consistent with prior literature linking endothelial activation and chronic inflammation to aneurysmal degeneration [40], though direct testing of pan-vascular mechanisms was beyond the scope of the present study. Subnetwork analysis further illustrates how candidates embed within these established modules: integrins cluster with collagens and laminins in ECM–receptor interaction and actin cytoskeleton regulation contexts, while SERPINE1, SERPINF2, and ITGA10 associate with coagulation and cytoskeletal remodeling processes [38,39], indicating that candidates both reinforce existing biological modules and extend known AAA biology into new functional axes.

### RNA- and microRNA-based contextual evidence for candidate proteins

**Fig. 7.**
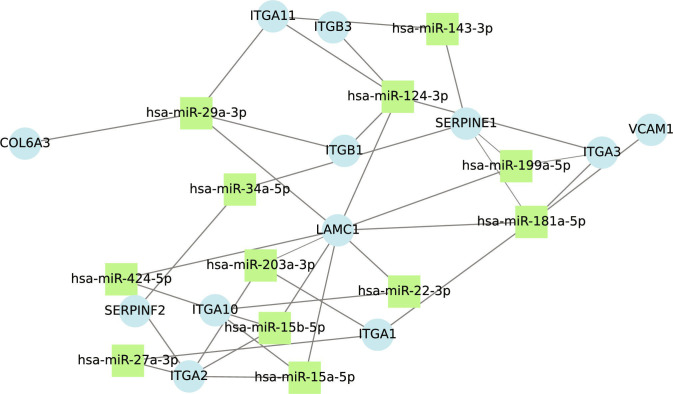
Interaction plot of miRNAs associated with AAA and their target genes/proteins, showing only the candidate proteins.

Several candidates show multilevel molecular support beyond protein-level predictions. ITGAV, VCAM1, SERPINE1, SERPINF2, ITGA1, ITGA2, and ITGB2 have been reported in AAA transcriptomic datasets, and a subset show predicted interactions with AAA-associated miRNAs (Fig. 7), suggesting regulatory involvement at both transcript and protein levels. Where RNA-level evidence is absent, protein-level analysis provides complementary insights not captured by transcriptomics alone [41].

### Broader aneurysm and dissection associations of candidate proteins

Several candidates have been independently reported in thoracic aortic aneurysm and aortic dissection studies [42], suggesting that the framework captures conserved pathological mechanisms across aneurysm phenotypes rather than AAA-specific biology alone. SERPINE1 [43–45] and SERPINF2 [46] operate at the intersection of protease regulation, inflammation, and ECM remodeling, processes shared across aneurysm types. LUM [47,48] has been proposed as a TAA and dissection biomarker, while ITGB3 [49–51], ITGB2 [52–54], ITGA1 [55], and VCAM1 [53,55–57] are implicated in cell adhesion, inflammatory activation, and SMC behavior across multiple aneurysm contexts.

These cross-disease associations are summarized comprehensively in Table S2. At the same time, proteins such as ITGA3 [58] and ITGA10 [59] have limited coverage in the aneurysm literature, placing them at the boundary of current knowledge. Their presence in the candidate set suggests that the graph-based approach highlights both well-established aneurysm-related proteins and those whose potential roles in aneurysm biology remain underexplored.

### Overlap of AAA candidate proteins with general cardiovascular disease pathways

The 19 candidates are broadly represented across major cardiovascular diseases. Most integrins in the candidate set appear in studies of atherosclerosis, myocardial infarction, peripheral arterial disease, stroke, and heart failure, reflecting their roles in cell–matrix interactions, immune cell recruitment, and mechanical regulation of the vessel wall [34,60]. ECM components including COL6A3, LUM, LAMC1, and LAMB1, and protease regulators SERPINE1, SERPINA5, and SERPINF2 show similar cross-disease patterns, each contributing to structural remodeling or protease balance in chronically injured arteries. A subset including ITGA10, ITGA11, and SERPINA5 have limited AAA-specific evidence, representing candidates where the model identifies underexplored vascular biology warranting targeted experimental evaluation.

### Clinical implications and digital health integration

The 19 candidate proteins represent a computationally prioritized set pending prospective validation before any clinical application can be considered, with translational potential spanning 3 axes.

First, several candidates have established or plausible plasma-detectable forms. SERPINE1 [43–45] and VCAM1 [53,56,57,61] have been quantified in plasma in cardiovascular disease contexts and represent near-term candidates for AAA plasma cohort validation [8,9,62]. Tissue-to-plasma biomarker translation has precedent in other diseases. PSA in prostate cancer, cardiac troponin in myocardial infarction, and CA-125 in ovarian cancer were all originally identified in tissue before successful plasma translation [63]. Dedicated prospective studies with validated detection platforms are required to determine whether similar translation is achievable for our candidates [60,62].

Second, several enzymatic candidates represent potential therapeutic targets. SERPINE1 inhibition has been explored in vascular disease [43,64,65] and the serpin family represents a tractable pharmacological target class [64]. Modulation of SERPINE1 and SERPINF2 activity in ECM remodeling and coagulation pathways [43–46] warrants dedicated experimental investigation.

Third, candidates with cell-surface expression, particularly integrins (ITGA1 [55], ITGA2 [32], ITGAV [66], ITGB1 [67,68], ITGB3 [49], and ITGB4) and VCAM1 [61], may be suitable for molecular imaging probe development. Integrin-targeted positron emission tomography (PET) tracers have clinical precedent in oncology [69], though AAA application remains speculative and requires preclinical validation.

Prospective validation is planned through the VASCULAID cohort at Amsterdam UMC (150+ cases enrolled), with plasma mass spectrometry proteomics as the primary validation platform. The current study should be understood as a hypothesis-generating framework defining a prioritized candidate list for this and future validation efforts [60,62]. Clinical actionability of the 19 candidates was assessed across 3 axes: prior detection in human AAA tissue by mass spectrometry or immunohistochemistry; reported differential expression between AAA and non-aneurysmal aortic tissue in at least one peer-reviewed study; and biological plausibility based on pathway enrichment in processes known to drive AAA pathophysiology. This evaluation is summarized in Table 3 and represents computational and literature-based assessment only; prospective experimental validation in patient cohorts is required before any clinical application can be considered.

### Limitations and future directions

Several methodological limitations warrant acknowledgement. First, the PU loss functions assume that labeled positives are selected completely at random (SCAR assumption), which is likely violated given that literature-derived AAA proteins are biased toward well-studied, abundant proteins. The direction of bias favors confirmation of established biology over novel discovery, though our prior sensitivity analysis demonstrates stable results across reasonable prior misspecification. Second, the enrichment-based refinement introduces circularity: enriched terms are defined jointly by the known AAA-associated proteins set and predicted candidates, systematically excluding proteins outside established biological axes. We address this by separately reporting 96 novel-module candidates, including 17 predicted by all 4 models, as an exploratory discovery set (Table S1). Third, the positive label set reflects the current published literature and may underrepresent low-abundance or tissue-restricted proteins; future iterations would benefit from curated, unbiased proteomics datasets.

Training set recall was uniformly higher than spy-set recall across all models (nnPU: training 0.94 vs. spy 0.78; PU-GNN: training 0.91 vs. spy 0.83), reflecting expected overfitting in the PU setting and underscoring the importance of spy-set evaluation for realistic performance assessment.

Future work should prioritize incorporating tissue-specific and single-cell PPI data to improve model granularity, advancing GNN interpretability methods, and integrating multiomics layers including transcriptomic, epigenomic, and metabolomic data. Experimental validation through mass spectrometry, enzyme-linked immunosorbent assay, single-cell transcriptomics, and spatial proteomics will be essential to confirm predictions, with functional investigations including CRISPR-based perturbation screens needed to establish causal roles for key candidates. Longitudinal tracking of biomarker dynamics in patient cohorts will be instrumental in moving toward personalized models of AAA progression. A user-friendly webserver is planned to facilitate community access to the framework and candidate list.

## Data Availability

The code and data used in this study are publicly available at https://github.com/RadhaKrishnaAyyalasomayajula/AAA-PU-GNN. Supplementary Data files S1 and S2 containing the full edge list and node properties are also provided as supplementary material to this article. The STRING protein interaction database is available at https://string-db.org and BioGRID at https://thebiogrid.org.
