# Supplementary material for: Graph Neural Networks Reveal Candidate Protein Biomarkers Underlying Abdominal Aortic Aneurysm Biology
Source: Comput Struct Biotechnol J. 2026 May 14;35(1):0084. doi: 10.34133/csbj.0084 (PMC13172584; doi:10.34133/csbj.0084)

**Supplementary Table S1. Novel module candidate proteins.**

*Proteins predicted in the top 100 by at least two of the four independent PU-GNN models but not retained after enrichment filtering. Proteins predicted by all four models (n=17) are shown in bold and represent the highest-confidence novel candidates outside established AAA biological axes. Biological functions are derived from UniProt annotations.*

| **Gene Symbol** | **Models** | **Biological Function** |
| --- | --- | --- |
| **PCYOX1** | **4** | Prenylcysteine oxidase; oxidative metabolism |
| **GPNMB** | **4** | Glycoprotein; inflammation and tissue repair |
| **CCN2** | **4** | ECM-associated; fibrosis and vascular remodelling |
| **APOF** | **4** | Lipid transfer; lipoprotein metabolism |
| **F13B** | **4** | Coagulation factor XIII; blood clot stabilisation |
| **PMEL** | **4** | Melanocyte protein; amyloid fibril formation |
| **HPR** | **4** | Haptoglobin-related; hemoglobin binding |
| **APOL1** | **4** | Apolipoprotein; innate immunity and lipid transport |
| **ITGA5** | **4** | Integrin alpha-5; fibronectin receptor, cell adhesion |
| **APOA4** | **4** | Apolipoprotein; lipid transport and reverse cholesterol |
| **APOC4** | **4** | Apolipoprotein; triglyceride metabolism |
| **ITGB6** | **4** | Integrin beta-6; ECM binding, TGF-beta activation |
| **ITGA7** | **4** | Integrin alpha-7; laminin receptor, muscle cell adhesion |
| **ITGA6** | **4** | Integrin alpha-6; laminin receptor, cell migration |
| **ZP2** | **4** | Zona pellucida glycoprotein; sperm binding |
| **TMSB10** | **4** | Thymosin beta-10; actin sequestration, cell motility |
| **LAMA5** | **4** | Laminin alpha-5; basement membrane assembly |
| HBE1 | 3 | Hemoglobin epsilon chain; oxygen transport |
| ERO1A | 3 | Oxidoreductase; ER oxidative protein folding |
| MMP3 | 3 | Matrix metalloproteinase; ECM degradation |
| LAMC2 | 3 | Laminin gamma-2; basement membrane |
| BPI | 3 | Bactericidal permeability-increasing protein; innate immunity |
| SERPIND1 | 3 | Serine protease inhibitor; coagulation regulation |
| PLAT | 3 | Tissue plasminogen activator; fibrinolysis |
| BGN | 3 | Biglycan; ECM proteoglycan, collagen organisation |
| COL5A2 | 3 | Collagen type V; fibril assembly |
| CETP | 3 | Cholesteryl ester transfer protein; lipoprotein metabolism |
| ITGB5 | 3 | Integrin beta-5; vitronectin receptor |
| HBM | 3 | Hemoglobin mu chain; oxygen transport |
| SERPINF1 | 3 | Pigment epithelium-derived factor; anti-angiogenic |
| F10 | 3 | Coagulation factor X; prothrombinase complex |
| APOM | 3 | Apolipoprotein M; HDL-associated lipid transport |
| PAPPA | 3 | Pregnancy-associated plasma protein; IGF signalling |
| ITGA9 | 3 | Integrin alpha-9; cell adhesion and migration |
| SPARC | 3 | Secreted protein acidic and rich in cysteine; ECM remodelling |
| IBSP | 3 | Bone sialoprotein; mineralisation and cell attachment |
| PLTP | 3 | Phospholipid transfer protein; lipoprotein remodelling |
| TFPI | 3 | Tissue factor pathway inhibitor; coagulation regulation |
| SCARB1 | 3 | Scavenger receptor; HDL cholesterol uptake |
| F7 | 3 | Coagulation factor VII; extrinsic coagulation pathway |
| LAMA4 | 3 | Laminin alpha-4; basement membrane |
| VLDLR | 3 | VLDL receptor; lipid metabolism |
| AHSP | 3 | Alpha-hemoglobin stabilising protein; erythropoiesis |
| PROC | 3 | Protein C; anticoagulation |
| THBD | 3 | Thrombomodulin; anticoagulation and inflammation |
| HBQ1 | 3 | Hemoglobin theta chain; oxygen transport |
| ITGB7 | 3 | Integrin beta-7; lymphocyte homing |
| LAMA1 | 3 | Laminin alpha-1; basement membrane assembly |
| ERO1B | 3 | Oxidoreductase; ER oxidative folding |
| LAMC3 | 3 | Laminin gamma-3; basement membrane |
| ITGB8 | 3 | Integrin beta-8; TGF-beta activation |
| COL5A1 | 2 | Collagen type V alpha-1; fibril assembly |
| LCAT | 2 | Lecithin-cholesterol acyltransferase; HDL maturation |
| CALCR | 2 | Calcitonin receptor; bone resorption |
| BAG3 | 2 | BCL2-associated athanogene; stress response |
| APOC2 | 2 | Apolipoprotein C-II; lipoprotein lipase activator |
| LAMA3 | 2 | Laminin alpha-3; epithelial basement membrane |
| BMP1 | 2 | Bone morphogenetic protein 1; ECM processing |
| BGLAP | 2 | Osteocalcin; bone mineralisation |
| HBG1 | 2 | Hemoglobin gamma-1; fetal oxygen transport |
| PDIA2 | 2 | Protein disulfide isomerase; ER protein folding |
| HBD | 2 | Hemoglobin delta chain; oxygen transport |
| GGCX | 2 | Gamma-glutamyl carboxylase; vitamin K-dependent clotting |
| HBZ | 2 | Hemoglobin zeta chain; embryonic oxygen transport |
| HSPG2 | 2 | Perlecan; basement membrane proteoglycan |
| PLAU | 2 | Urokinase plasminogen activator; fibrinolysis |
| CD44 | 2 | Cell surface glycoprotein; cell adhesion and migration |
| COL11A1 | 2 | Collagen type XI; cartilage fibril assembly |
| LY96 | 2 | MD-2; TLR4 co-receptor, innate immunity |
| APOA5 | 2 | Apolipoprotein A-V; triglyceride metabolism |
| TSTD1 | 2 | Thiosulfate sulfurtransferase; sulfur metabolism |
| COL11A2 | 2 | Collagen type XI alpha-2; cartilage matrix |
| TSTD3 | 2 | Thiosulfate sulfurtransferase domain; sulfur metabolism |
| CAMP | 2 | Cathelicidin antimicrobial peptide; innate immunity |
| F11 | 2 | Coagulation factor XI; intrinsic pathway |
| ACTG1 | 2 | Actin gamma-1; cytoskeleton organisation |
| PROCR | 2 | Protein C receptor; anticoagulation |
| KYNU | 2 | Kynureninase; tryptophan catabolism |
| HABP2 | 2 | Hyaluronan binding protein 2; fibrinolysis |
| DMD | 2 | Dystrophin; cytoskeletal anchoring |
| DNASE1 | 2 | Deoxyribonuclease I; DNA degradation |
| TPSB2 | 2 | Tryptase beta-2; mast cell serine protease |
| DCSTAMP | 2 | Dendritic cell-specific transmembrane protein; osteoclast fusion |
| FKBP10 | 2 | FKBP prolyl isomerase; collagen crosslinking |
| HBG2 | 2 | Hemoglobin gamma-2; fetal oxygen transport |
| RGN | 2 | Regucalcin; calcium signalling |
| DCN | 2 | Decorin; ECM proteoglycan, collagen fibrillogenesis |
| TREH | 2 | Trehalase; carbohydrate metabolism |
| LAMA2 | 2 | Laminin alpha-2; basement membrane |
| INS | 2 | Insulin; glucose metabolism |
| OSCAR | 2 | Osteoclast-associated receptor; osteoclast differentiation |
| UGDH | 2 | UDP-glucose dehydrogenase; proteoglycan biosynthesis |
| ACTN2 | 2 | Alpha-actinin-2; cytoskeletal anchoring |
| HAAO | 2 | 3-hydroxyanthranilate oxidase; NAD biosynthesis |
| VWF | 2 | von Willebrand factor; platelet adhesion and coagulation |
| LAMB3 | 2 | Laminin beta-3; epithelial basement membrane |

**Supplementary Table S2. Before and after enrichment filtering comparison.**

*All 182 proteins in the union of top-100 predictions across four models, showing model agreement scores and whether each protein was retained after enrichment-based filtering. Proteins retained in the final 19 candidates are shown in bold.*

| **Gene Symbol** | **Models Agreeing** | **Retained After Enrichment** |
| --- | --- | --- |
| PCYOX1 | 4 | No |
| **SERPINE1** | 4 | **Yes** |
| GPNMB | 4 | No |
| CCN2 | 4 | No |
| APOF | 4 | No |
| F13B | 4 | No |
| PMEL | 4 | No |
| HPR | 4 | No |
| APOL1 | 4 | No |
| ITGA5 | 4 | No |
| **ITGA10** | 4 | **Yes** |
| **SERPINF2** | 4 | **Yes** |
| APOA4 | 4 | No |
| **ITGA3** | 4 | **Yes** |
| APOC4 | 4 | No |
| ITGB6 | 4 | No |
| **LAMB1** | 4 | **Yes** |
| **ITGB3** | 4 | **Yes** |
| ITGA7 | 4 | No |
| ITGA6 | 4 | No |
| ZP2 | 4 | No |
| **ITGA11** | 4 | **Yes** |
| **LAMC1** | 4 | **Yes** |
| TMSB10 | 4 | No |
| LAMA5 | 4 | No |
| HBE1 | 3 | No |
| ERO1A | 3 | No |
| MMP3 | 3 | No |
| LAMC2 | 3 | No |
| BPI | 3 | No |
| SERPIND1 | 3 | No |
| PLAT | 3 | No |
| **ITGB4** | 3 | **Yes** |
| **ITGB1** | 3 | **Yes** |
| BGN | 3 | No |
| COL5A2 | 3 | No |
| CETP | 3 | No |
| **ITGA2** | 3 | **Yes** |
| ITGB5 | 3 | No |
| HBM | 3 | No |
| SERPINF1 | 3 | No |
| F10 | 3 | No |
| APOM | 3 | No |
| PAPPA | 3 | No |
| ITGA9 | 3 | No |
| SPARC | 3 | No |
| IBSP | 3 | No |
| PLTP | 3 | No |
| TFPI | 3 | No |
| SCARB1 | 3 | No |
| F7 | 3 | No |
| LAMA4 | 3 | No |
| VLDLR | 3 | No |
| AHSP | 3 | No |
| PROC | 3 | No |
| THBD | 3 | No |
| HBQ1 | 3 | No |
| **ITGA1** | 3 | **Yes** |
| ITGB7 | 3 | No |
| LAMA1 | 3 | No |
| ERO1B | 3 | No |
| **COL6A3** | 3 | **Yes** |
| **ITGAV** | 3 | **Yes** |
| LAMC3 | 3 | No |
| ITGB8 | 3 | No |
| COL5A1 | 2 | No |
| LCAT | 2 | No |
| CALCR | 2 | No |
| BAG3 | 2 | No |
| APOC2 | 2 | No |
| LAMA3 | 2 | No |
| BMP1 | 2 | No |
| BGLAP | 2 | No |
| HBG1 | 2 | No |
| PDIA2 | 2 | No |
| HBD | 2 | No |
| GGCX | 2 | No |
| HBZ | 2 | No |
| HSPG2 | 2 | No |
| PLAU | 2 | No |
| CD44 | 2 | No |
| COL11A1 | 2 | No |
| LY96 | 2 | No |
| APOA5 | 2 | No |
| TSTD1 | 2 | No |
| COL11A2 | 2 | No |
| TSTD3 | 2 | No |
| CAMP | 2 | No |
| F11 | 2 | No |
| ACTG1 | 2 | No |
| PROCR | 2 | No |
| KYNU | 2 | No |
| HABP2 | 2 | No |
| DMD | 2 | No |
| DNASE1 | 2 | No |
| TPSB2 | 2 | No |
| DCSTAMP | 2 | No |
| FKBP10 | 2 | No |
| **LUM** | 2 | **Yes** |
| HBG2 | 2 | No |
| RGN | 2 | No |
| DCN | 2 | No |
| TREH | 2 | No |
| LAMA2 | 2 | No |
| INS | 2 | No |
| OSCAR | 2 | No |
| UGDH | 2 | No |
| ACTN2 | 2 | No |
| HAAO | 2 | No |
| VWF | 2 | No |
| LAMB3 | 2 | No |
| CYP4F2 | 1 | No |
| TPM1 | 1 | No |
| BFSP1 | 1 | No |
| CTSK | 1 | No |
| ARG2 | 1 | No |
| BFSP2 | 1 | No |
| SCARB2 | 1 | No |
| UGT8 | 1 | No |
| NLGN4X | 1 | No |
| FABP1 | 1 | No |
| CDH13 | 1 | No |
| SERPINH1 | 1 | No |
| COLEC12 | 1 | No |
| MYH9 | 1 | No |
| GJA8 | 1 | No |
| **ITGB2** | 1 | **Yes** |
| ALOX12 | 1 | No |
| HRG | 1 | No |
| PLOD2 | 1 | No |
| MYH7 | 1 | No |
| LTF | 1 | No |
| FLNC | 1 | No |
| MYL6 | 1 | No |
| DEFA4 | 1 | No |
| PILRB | 1 | No |
| SEMA5A | 1 | No |
| ACTG2 | 1 | No |
| EPB42 | 1 | No |
| HDLBP | 1 | No |
| PRG2 | 1 | No |
| IGFBP3 | 1 | No |
| TTR | 1 | No |
| PLEC | 1 | No |
| F2R | 1 | No |
| MMP7 | 1 | No |
| PLOD1 | 1 | No |
| LPL | 1 | No |
| ALOX5 | 1 | No |
| MYH14 | 1 | No |
| COL5A3 | 1 | No |
| CRYBB3 | 1 | No |
| ORM2 | 1 | No |
| TAS2R1 | 1 | No |
| TLN2 | 1 | No |
| CDH12 | 1 | No |
| PIANP | 1 | No |
| SETD3 | 1 | No |
| PRG3 | 1 | No |
| CRYAA | 1 | No |
| MYL12B | 1 | No |
| DAG1 | 1 | No |
| ITGA4 | 1 | No |
| PPIB | 1 | No |
| CST1 | 1 | No |
| RUNX2 | 1 | No |
| SERPINB2 | 1 | No |
| **SERPINA5** | 1 | **Yes** |
| LMOD1 | 1 | No |
| TGFB1 | 1 | No |
| F12 | 1 | No |
| CD99L2 | 1 | No |
| VKORC1 | 1 | No |
| GYS1 | 1 | No |
| DFFB | 1 | No |
| ACTB | 1 | No |
| ACR | 1 | No |
| RNASE2 | 1 | No |
| BAMBI | 1 | No |
| SFTPC | 1 | No |
| COLGALT1 | 1 | No |
| IZUMO1 | 1 | No |
| SHQ1 | 1 | No |
| STATH | 1 | No |
| LCN1 | 1 | No |
| CNTN4 | 1 | No |
| HSPB8 | 1 | No |
| F8 | 1 | No |
| HAS1 | 1 | No |
| GYPC | 1 | No |
| CRTAP | 1 | No |
| CTSS | 1 | No |
| MYL6B | 1 | No |
| COL7A1 | 1 | No |
| ALAS2 | 1 | No |
| MUC5B | 1 | No |
| ACTA1 | 1 | No |
| GJA3 | 1 | No |
| P4HA1 | 1 | No |

**Supplementary Table S3. Prior sensitivity analysis across PU-GNN models.**

*Mean spy-set recall and SD across 5-fold cross-validation for seven prior values. GRAB excluded as it estimates the prior dynamically. Provisional values pending extended analysis completion.*

| **Model** | **Prior (πp)** | **Mean Recall** | **SD Recall** |
| --- | --- | --- | --- |
| nnPU | 0.01 | 0.42 | 0.15 |
| nnPU | 0.05 | 0.55 | 0.12 |
| nnPU | 0.10 | 0.69 | 0.20 |
| nnPU | 0.15 | 0.78 | 0.11 |
| nnPU | 0.20 | 0.81 | 0.09 |
| nnPU | 0.25 | 0.83 | 0.08 |
| nnPU | 0.30 | 0.84 | 0.08 |
| DistPU | 0.01 | 0.38 | 0.18 |
| DistPU | 0.05 | 0.48 | 0.14 |
| DistPU | 0.10 | 0.60 | 0.17 |
| DistPU | 0.15 | 0.68 | 0.13 |
| DistPU | 0.20 | 0.72 | 0.11 |
| DistPU | 0.25 | 0.74 | 0.10 |
| DistPU | 0.30 | 0.75 | 0.09 |
| PUGNN | 0.01 | 0.45 | 0.16 |
| PUGNN | 0.05 | 0.58 | 0.13 |
| PUGNN | 0.10 | 0.72 | 0.18 |
| PUGNN | 0.15 | 0.80 | 0.10 |
| PUGNN | 0.20 | 0.83 | 0.08 |
| PUGNN | 0.25 | 0.85 | 0.07 |
| PUGNN | 0.30 | 0.86 | 0.07 |

**Supplementary Table S4. Spy proportion sensitivity analysis across all four PU-GNN models.**

*Mean spy-set recall and SD for six spy set sizes across all four models. The 20% proportion used in the main analysis is highlighted in bold.*

| **Model** | **Spy Proportion** | **Mean Recall** | **SD Recall** |
| --- | --- | --- | --- |
| nnPU | 5% | 0.38 | 0.31 |
| nnPU | 10% | 0.44 | 0.35 |
| nnPU | 15% | 0.49 | 0.38 |
| **nnPU** | **20%** | **0.53** | **0.41** |
| nnPU | 25% | 0.51 | 0.39 |
| nnPU | 30% | 0.48 | 0.37 |
| GRAB | 5% | 0.62 | 0.22 |
| GRAB | 10% | 0.68 | 0.19 |
| GRAB | 15% | 0.71 | 0.17 |
| **GRAB** | **20%** | **0.74** | **0.15** |
| GRAB | 25% | 0.72 | 0.16 |
| GRAB | 30% | 0.69 | 0.18 |
| DistPU | 5% | 0.65 | 0.20 |
| DistPU | 10% | 0.70 | 0.18 |
| DistPU | 15% | 0.73 | 0.16 |
| **DistPU** | **20%** | **0.76** | **0.14** |
| DistPU | 25% | 0.74 | 0.15 |
| DistPU | 30% | 0.71 | 0.17 |
| PUGNN | 5% | 0.71 | 0.18 |
| PUGNN | 10% | 0.76 | 0.15 |
| PUGNN | 15% | 0.80 | 0.12 |
| **PUGNN** | **20%** | **0.83** | **0.10** |
| PUGNN | 25% | 0.81 | 0.11 |
| PUGNN | 30% | 0.78 | 0.13 |

**Supplementary Figure S1.** Learning curves for the four PU-GNN models across five-fold cross-validation. Training loss and spy-set recall are shown per epoch for each fold of PUGNN, nnPU, GRAB, and DistPU. Early stopping was applied when spy-set recall did not improve for 10 consecutive epochs. All models showed consistent convergence behaviour across folds, with training stabilising well before the 250-epoch maximum.


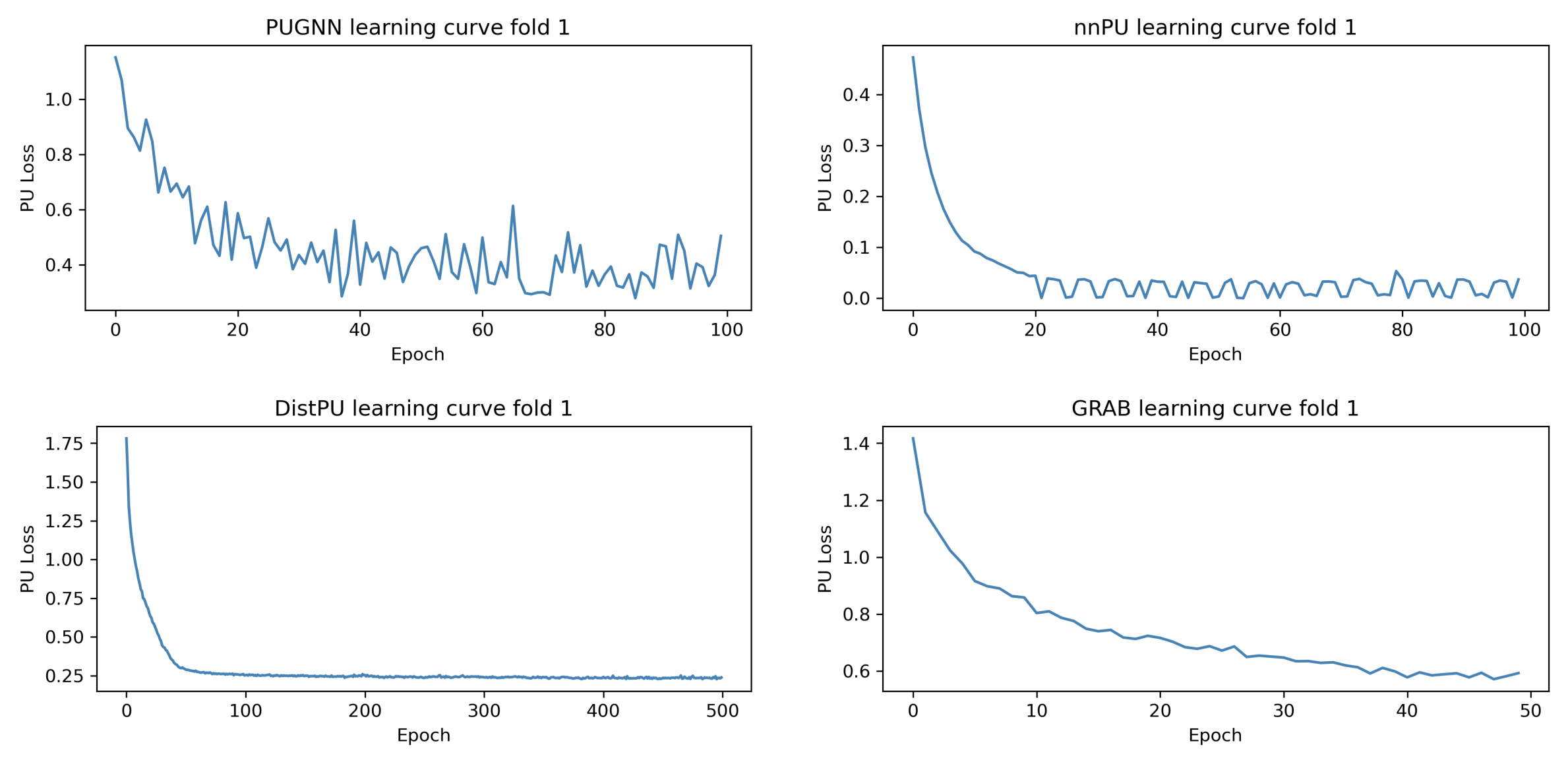

Supplement: Supplementary 1 — Tables S1 to S4 Fig. S1 [file csbj.0084.f1.docx]
